# Current status and perspectives of genome editing technology for microalgae

**DOI:** 10.1186/s13068-017-0957-z

**Published:** 2017-11-14

**Authors:** Seungjib Jeon, Jong-Min Lim, Hyung-Gwan Lee, Sung-Eun Shin, Nam Kyu Kang, Youn-Il Park, Hee-Mock Oh, Won-Joong Jeong, Byeong-ryool Jeong, Yong Keun Chang

**Affiliations:** 1 Advanced Biomass Research and Development Center (ABC), 291 Daehak-ro, Yuseong-gu, Daejeon, 34141 Republic of Korea; 2 Department of Chemical and Biomolecular Engineering, Korea Advanced Institute of Science and Technology (KAIST), 291 Daehak-ro, Yuseong-gu, Daejeon, 34141 Republic of Korea; 3 Plant Systems Engineering Research Center, Korea Research Institute of Bioscience and Biotechnology (KRIBB), 125 Gwahak-ro, Yuseong-gu, Daejeon, 34141 Republic of Korea; 4 Cell Factory Research Center, Korea Research Institute of Bioscience and Biotechnology (KRIBB), 125 Gwahak-ro, Yuseong-gu, Daejeon, 34141 Republic of Korea; 5 LG Chem, 188 Munji-ro, Yuseong-gu, Daejeon, 34122 Republic of Korea; 6 Department of Biological Sciences, Chungnam National University, Daejeon, 34134 Republic of Korea

**Keywords:** Genetic engineering, Microalgae, Genome editing, CRISPR/Cas9, Biofuels, GMO conflicts

## Abstract

Genome editing techniques are critical for manipulating genes not only to investigate their functions in biology but also to improve traits for genetic engineering in biotechnology. Genome editing has been greatly facilitated by engineered nucleases, dubbed molecular scissors, including zinc-finger nuclease (ZFN), TAL effector endonuclease (TALEN) and clustered regularly interspaced palindromic sequences (CRISPR)/Cas9. In particular, CRISPR/Cas9 has revolutionized genome editing fields with its simplicity, efficiency and accuracy compared to previous nucleases. CRISPR/Cas9-induced genome editing is being used in numerous organisms including microalgae. Microalgae have been subjected to extensive genetic and biological engineering due to their great potential as sustainable biofuel and chemical feedstocks. However, progress in microalgal engineering is slow mainly due to a lack of a proper transformation toolbox, and the same problem also applies to genome editing techniques. Given these problems, there are a few reports on successful genome editing in microalgae. It is, thus, time to consider the problems and solutions of genome editing in microalgae as well as further applications of this exciting technology for other scientific and engineering purposes.

## Background

Targeted genome modifications are crucial for genetic analyses and genetic engineering in all aspects of biology and related biotechnological fields. Different from random integration of cloned genes for overexpression, specific alterations of the eukaryotic genome have been great challenges for all biologists and biotechnologists. Gene targeting (GT) was initially developed in recombinogenic lower eukaryotes by introducing a homologous transgene into the cell, and by utilizing homologous recombination (HR), scientists were able to knockout or replace genes of interest [1]. GT has been successfully demonstrated in animals [2, 3] and plants [4]. However, GT in these higher organisms has been very difficult, in part, because they are not recombinogenic [5]. Newly developed techniques, including genome editing techniques, have bypassed this hurdle by engineered nucleases, dubbed “molecular scissors,” and the subsequent repair of DNA strand breaks results in mutations or replacements of the genes of interest [6].

Engineered nucleases include zinc-finger nucleases (ZFNs), transcription activator-like effector nucleases (TALENs), and clustered regularly interspaced palindromic sequences (CRISPR)/CRISPR-associated protein 9 (Cas9) [7]. These three, in particular CRISPR/Cas9, will be described for microalgal genome editing in this review even though there have been other nucleases including meganucleases and group II intron-based targetrons adopted for genome editing in other organisms [8]. These sequence-specific nucleases have enabled researchers to cleave genomic DNA and to obtain mutations of a gene resulting from faulty repair of the cleaved DNA.

Microalgae have emerged as important platforms for the production of biofuels and other biomolecules, and genetic engineering of microalgae is, thus, one of the fastest growing biotechnology fields [9]. In addition to overexpression of genes of interest, genome editing is essential for the suppression of genes interfering with the production of target molecules. However, progress in this field has been hampered by multiple layers of difficulties inherent to microalgae. This review will describe what has been achieved in microalgal genome editing and examine in detail the problems associated with microalgal genome editing and suggest possible solutions. Genome editing has many applications that have been shown in other organisms, and the possibility of these applications in microalgae will also be evaluated. Because the overexpression of genes shares the same technical problems, we will also include a brief description of microalgal transformation technology.

## Introduction to genetic engineering

Genetic engineering, by definition, requires the delivery of genetic material to the genome resulting in genetic modifications. Since reports of successful transfection of animal cells with isolated viral DNAs in the early 1970s [10, 11], it took more than 10 years to achieve the transformation of plants using Agrobacteria that are capable of transforming plants in nature [12]. As usual, transformation of the model microalga *Chlamydomonas* came much later in the late 1980s [13, 14]. The long delay in achieving microalgae transformation was not simply due to a smaller microalgal community but also to technical problems inherent to microalgae. The same delay was seen for genome editing techniques in microalgae. As pointed out lately, successful genome editing should be based on solid transformation techniques for an organism [15]. We, thus, summarize the historical perspectives of plant and microalgal transformation, which can provide clues to genome editing in microalgae.

Transformation of plants lagged behind animals probably due to the presence of the protective cell wall, and initial attempts of a workaround with protoplasts did not provide much success [16]. Instead, plant geneticists developed alternative techniques using the natural transformation machinery of Agrobacteria, which circumvented the removal of the cell wall [12]. In addition to Agrobacteria, particle bombardment also gained popularity, which can avoid the host range limitations of Agrobacteria [17]. Later on, the removal of the plant cell wall improved, and transformation of protoplasts via polyethylene glycol (PEG) or electroporation was also established in plants [18, 19].

Transformation of the model microalga *Chlamydomonas reinhardtii* was achieved by the transformation techniques used for plants, which was done for nuclear [13, 14] and chloroplast genomes [20]. Later, transformation by glass bead agitation was uniquely developed for *C. reinhardtii*, where protoplast cells were agitated vigorously with glass beads [21]. These pioneering works led to the development of the transformation toolbox in *Chlamydomonas* and other microalgae summarized in Table 1. In general, delivery of genetic material in microalgae is considered much less efficient compared to that of plants. On the other hand, once transformants are obtained, microalgae are mostly single cells and can be maintained somatically, not requiring the tedious (and recalcitrant in some species) regeneration steps in plants [15].

**Table 1:** Development of the transformation toolbox in microalgae

Algal strain	Delivery	Marker	Selection/stable integration/comments	References
*Chlamydomonas reinhardtii*				
*C. reinhardtii* (*arg7*-)	Particle bombardment	*ARG7*	Growth in arginine free medium/southern blot/correlation of the genetic and molecular maps of the ARG7 locus	[14]
*C. reinhardtii* (*nit1*-*305*)	Particle bombardment	*NIT1*	Growth in the presence of nitrate/southern blot/complementation with NR deficient mutants	[153]
*C. reinhardtii* Fud44 (OEE1-)	Particle bombardment	*OEE1*	Photoautotrophic growth/southern blot/complementation with OEE1-deficient mutants	[154]
A54-e18 (*ac17 nitl*-*Δ1 sr1*) and J9 (*cw15 nit1*-*305*)	Glass beads agitation	*CRY1*-*1*	Resistance to emetine/heritable integration and southern blot/dominant selectable marker gene	[21]
*C. reinhardtii* (*nic7*-)	Glass beads agitation	*nic*-*7*	Resistance to 3-acetylpyridine/southern blot/dominant selectable marker gene	[155]
*C. reinhardtii* *363* (*arg7*-*8* cw_d_)	Glass beads agitation	*Sh ble*	Resistance to phleomycin/southern and western blots/first inheritable expression of a foreign gene in *C. reinhardtii*	[22]
*C. reinhardtii* CC-124	Particle bombardment	*aadA*	Resistance to spectinomycin/southern, northern and western blots/analyses of mRNA expression and stability of *aadA*	[156]
*C. reinhardtii* 302 (cw15 arg7-8) and A (cw15 arg7)	Glass beads agitation	*aphVIII*	Resistance to paromomycin/southern, northern and western blots/expression of the *aphVIII* in *C. reinhardtii* in combination with different promoters from *rbcS2*, *hsp70A* and chlamyopsin	[157]
*C. reinhardtii* *302 cw15 arg2* and CC-124	Glass beads agitation	*aphVII*	Resistance to hygromycin B/RT-PCR and southern blot/second heterologous marker	[158]
*C. reinhardtii* CC-125, CC-425 and *cw*_*d*_-*ARG*	Glass beads and electroporation	*ALS*	Resistance to SMM/southern blot/strong promoter from *RbcS2* for proper expression of *ALS*	[159]
*Chlorella*				
*C. sorokiniana* (NR-UV9-5)	Particle bombardment	*NIT1*	Growth in the presence of nitrate/southern blot and RNase protection assay/rescue of nitrate reductase deficient *C. sorokiniana* mutant	[40]
*C. vulgaris*	Electroporation	*hph*	Resistance to hygromycin B/southern blot/dominant selectable marker gene	[160]
*C. vulgaris* C-27 *C. sorokiniana*, ATCC-22521	PEG-mediated transformation	*Neo* ^*r*^	Resistance to G418 (geneticin)/unstable integration/production of human growth hormone	[27]
*C. ellipsoids* KMCC C-20	PEG-mediated transformation	*Sh ble*	Resistance to phleomycin/southern and western blots/production of flounder growth hormone for feed	[29]
*C. vulgaris*	Electroporation	*CAT*	Resistance to chloramphenicol/PCR/heterologous promoter of *NR* from diatom	[161]
*C. zofingiensis* ATCC 30412	Particle bombardment and electroporation	*PDS*-L516F	Resistance to norflurazon/PCR and southern blot/increased production of carotenoids	[162]
*C. ellipsoidea*	Electroporation	*Npt*	Resistance to G418/PCR, RT-PCR and southern blot/heterologous expression of GmDof4 from soybean for increased lipid	[163]
*C. vulgaris* CBS 15-2075	PEG-mediated	*NptII*	Resistance to G418/southern blot/expression of EGFP	[26]
*Phaeodactylum tricornutum*				
*P. tricornutum* Strain 646	Particle bombardment	*Sh ble*	Resistance to zeocin/southern, northern and western blots/transformation toolbox for *P. tricornutum*	[164]
*P. tricornutum* Strain 646	Particle bombardment	*NptII*	Resistance to neomycin/PCR and western blot/transformation toolbox for *P. tricornutum*	[165]
*P. tricornutum* CCMP632	Particle bombardment	*Sh ble*	Resistance to phleomycin/PCR, RT-PCR, southern and western blots/RNA silencing by anti-sense or inverted repeats	[166]
*P. tricornutum* UTEX 646	Particle bombardment	*Sh ble*	Resistance to zeocin/none/increased DHA contents by heterologous Δ5-elongase	[167]
*Nannochloropsis*				
*N. oceanica* W2J3B	Electroporation	*Sh ble*	Resistance to zeocin/PCR/gene targeting of nitrate reductase and nitrite reductase genes	[116]
*N. oceanica* CCMP1779	Electroporation	*aphVII*	Resistance to hygromycin B/southern blot/sequencing genomic DNA and functional annotation in *N. oceanica*	[24]
*N. gaditana* CCMP526	Electroporation	*Sh ble*	Resistance to zeocin/PCR and southern blot/transformation toolbox for *N. gaditana*	[168]
*N. salina* CCMP 1776	Particle bombardment	*Sh ble*	Resistance to zeocin/PCR and western blot/stable expression of foreign genes	[169]
*N. salina* CCMP 1776	Particle bombardment	*Sh ble*	Resistance to zeocin/PCR, RT-PCR, southern and western blots/overexpression of NsbHLH2 for increased lipid productivity	[23]
*Dunaliella*				
*D. salina*	Electroporation	*CAT*	Resistance to chloramphenicol/PCR, RT-PCR, southern, northern and western blots/stable expression of foreign genes	[170]
*D. salina*	Electroporation	*Sh ble*	Resistance to zeocin/PCR, RT-PCR and southern blot/transformation toolbox for *D. salina*	[171]
*D. viridis* B14 (*NIA1*-)	Electroporation	*NIA1*	Growth in the presence of nitrate salt/RT-PCR and southern blot/complementation with NR deficient mutants	[172]
*D. salina*	Electroporation	*CAT*	Resistance to chloramphenicol/PCR and RT-PCR/RNA silencing by RNAi in *D. salina*	[173]
*D. salina* 19/18	Particle bombardment	*CAT*	Resistance to chloramphenicol/PCR and southern blot/increased total lipid content by 12% through endogenous expression of *ME/AccD*	[174]
*Haematococcus pluvialis*				
*H. pluvialis* NIES-144	Particle bombardment	*PDS*-L504R	Resistance to norflurazon/southern, northern and western blots/production of astaxanthin	[175]
*H. pluvialis* SAG 19-a	*Agrobacterium*-mediated transformation	*hph*	Resistance to hygromycin/PCR and southern blot/transformation toolbox for *H. pluvialis*	[176]
*H. pluvialis* SAG 34-1a	*Agrobacterium*-mediated transformation	*hph*	Resistance to hygromycin/PCR and southern blot/overexpression of *bkt* for increased carotenoids and astaxanthin production	[177]

*aadA*, aminoglycoside 3′-adenyltransferase; *ALS*, acetolactate synthase; *aphVII*, aminoglycoside phosphotransferase; *aphVIII*, aminoglycoside 3′-phosphotransferase; *ARG7*, argininosuccinate lyase; *AccD*, acetyl CoA carboxylase; *bkt*, beta carotene ketolase; *CAT*, chloramphenicol acetyltransferase; *CRY1*-*1*, ribosomal protein S14; DHA, docosahexaenoic acid; *hph*, hygromycin phosphotransferase; *ME*, malic enzyme; Neo^r^, neomycin phosphotransferase; *nic*-*7*, quinolinate synthetase; *NIT1*, *NIA1*, nitrate reductase; *Npt*, neomycin phosphotransferase; *OEE1*, oxygen-evolving enhancer protein1; *PDS*, phytoene desaturase; *Sh ble*, phleomycin binding protein; SMM, sulfometuron methyl

The development of the transformation toolbox in microalgae, excluding *Chlamydomonas*, presented another layer of difficulties mainly due to the paucity of selection markers that have been developed for plants and other microbes (Table 1). This is probably caused by their evolutionary divergence, where their cellular machineries have been differentiated so much that existing herbicides and antibiotics cannot be used for selection purposes. This is particularly true for the model “industrial microalgae” *Nannochloropsis* that are tolerant to most antibiotics and herbicides, which leaves only phleomycin and its derivative Zeocin for selection by the *ble* gene from *Streptoalloteichus hindustanus* (*Shble*) [22, 23]. Exceptionally, *N. oceanica* CCMP1779 is sensitive to hygromycin that is selectable with *aphVII* [24]. However, green algae sharing lineages with plants offers more options of selection, including hygromycin and other herbicides and antibiotics, similar to that of plants [25].

Microalgal transformation still suffers from an extremely low transformation efficiency, even compared to plants, which calls for drastically improved transformation techniques. Lately, cell wall removal and PEG-mediated protoplast transformation have been reported in *Chlorella* and *Cyanidioschyzon* [26–29]. PEG-mediated protoplast transformation in plants achieves a higher transformation efficiency without the concerns of the host range of Agrobacteria or expensive equipment [30]. *Chlorella* is gaining popularity in many global consortia of algal biofuel production, and it is, thus, interesting to see how this technique develops not only in *Chlorella* but also in other microalgae. Another technique that can be considered is bacterial conjugation for delivery of episomal vectors into microalgal cells, which is claimed to be more efficient than conventional transformation techniques in diatoms [31]. Optimized techniques of these might provide solutions for the current problems of delivery techniques in microalgae, which may be used for genome editing in microalgae.

Development of the transformation toolboxes also provided opportunities for reverse genetic techniques in microalgae, facilitated by the genomic sequencing of *C. reinhardtii* and other microalgae [24, 32]. These include RNA interference (RNAi) and artificial microRNAs (amiRNAs), based on the findings that microalgae are capable of RNA silencing by small interfering RNAs (siRNA) and microRNAs (miRNAs) in *C. reinhardtii* and in other microalgae [24, 33–38]. In addition to the RNA-based knockdown techniques, gene targeting via HR was also introduced in *Chlamydomonas* and *Chlorella* [39, 40]; however, these results were not reproducible probably due to the non-recombinogenic nature of microalgae. Similar difficulties were also observed in most of higher eukaryotes including animals and plants [2–4] because gene targeting was originally developed in recombinogenic yeasts [1]. Difficulties and/or inefficiencies of the above reverse genetic techniques called for a more efficient and precise modification of DNA, which led to genome editing, also known as genome engineering, using engineered nucleases. This review will further discuss genome editing, mainly focusing on microalgae, including difficulties and possible solutions.

## Genome editing using engineered nucleases

**Fig. 1:**
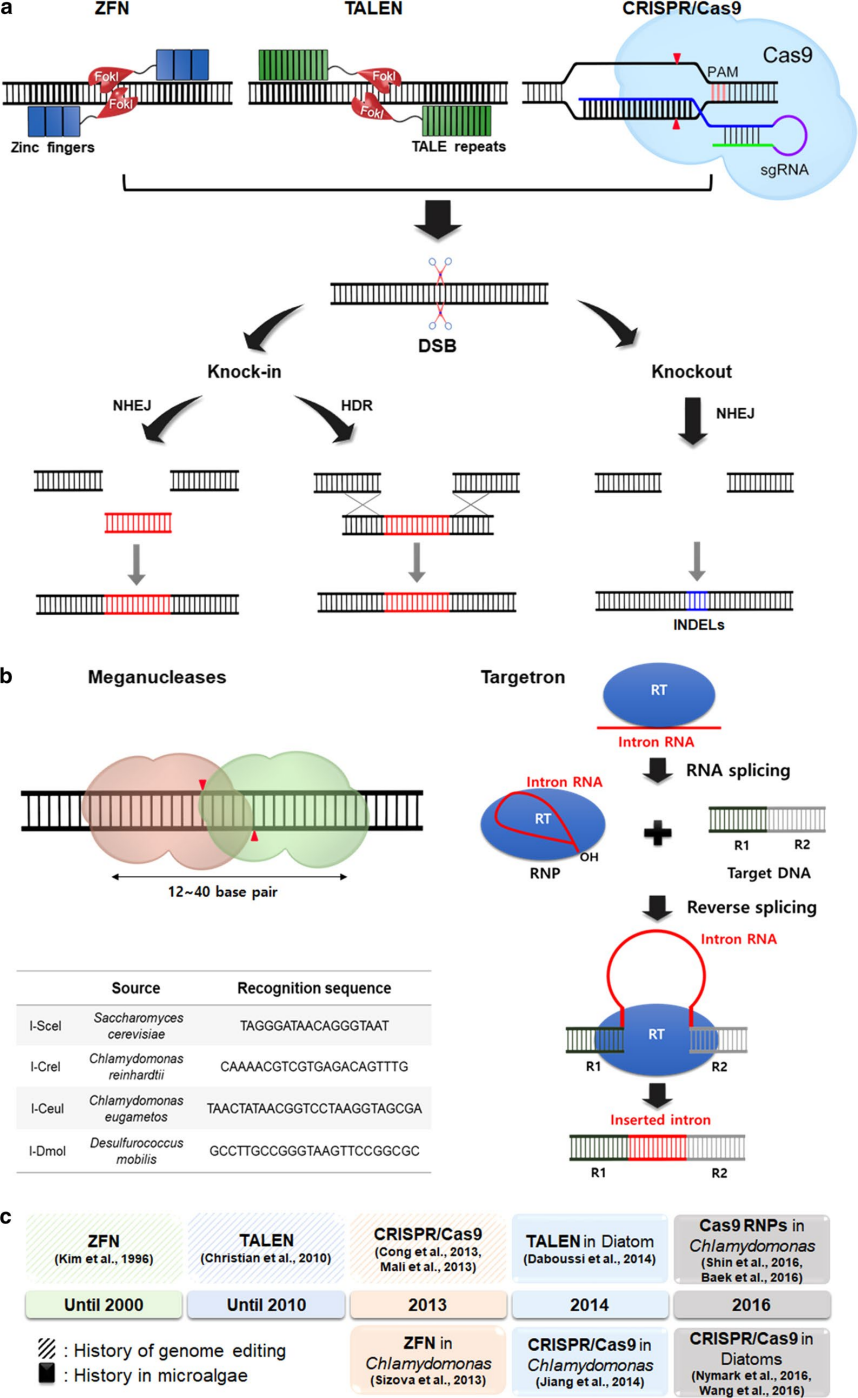
Summary of genome editing techniques using engineered nucleases. The first two nucleases are made by a fusion of a zinc-finger protein and TALE to the restriction enzyme *Fok*I, producing ZFN and TALEN, respectively (**a**). In contrast, Cas9 contains nuclease domains for the cleavage of DNA and RNA binding domains for the guide RNAs, which offer simplicity and better accuracy compared to the predecessors. All three nucleases produce DSBs, and INDELs can be produced via error-prone DNA repair NHEJ. When donor DNAs (red) are provided, knock-in events can be produced via either NHEJ or HDR. Other types of nucleases were summarized (**b**), including meganucleases and targetrons. Timeline of the major nucleases and their use in genome editing were summarized in **c**. Their first reports are shown in shades, and those of microalgal genome editing are shown in solid boxes

Genome editing uses recombinant nucleases engineered to recognize and cleave specific sequences in the genome, resulting in double strand breaks (DSBs). DSBs are repaired mostly by a homology-independent and error-prone DNA repair mechanism, called non-homologous end joining (NHEJ), resulting in mutations at the cleavage site [41–46]. Nucleases include ZFN, TALEN and CRISPR/Cas9 as summarized in Fig. 1a, and Table 2 lists representative cases of genome editing in plants and animals and all known cases of microalgal genome editing. Unfortunately, attempts of genome editing in microalgae have had limited success with only a handful of reports. Other endonucleases have been used for genome editing in other organisms, including meganucleases and group II intron-based targetrons as summarized in Fig. 1b [8, 47, 48]. It would be interesting to find out how these nucleases work in microalgae.

**Table 2:** Genome editing in microalgae and other organisms

Organism	Delivery/nuclease	Gene/marker	Comments	References
Animals				
CHO-S cells (11619-012)	Lipofectamine 2000 transfection ZFN	DHFR	Identification of biallelic knockouts after a single transient ZFN treatment	[57]
SKOV3 cells ATCC	FuGENE HD transfection reagent TALEN	EGFP and DsRed	Development of TALENs in mammalian cells	[59]
Mouse cells NIH3T3	Microinjection or Lipofectamine 2000 CRISPR/Cas9	EGFP and *ApoE*	Improvement of bi-allelic modification by dual sgRNAs	[178]
Human cell lines MCF-7, NSCLC and A549	Lipofectamine 2000 transfection CRISPR/Cas9	Mitochondrial GTPase Mitofusin-2/GFP	Identification of role MFN2 in human cells	[179]
H9 human ES cells and other cell lines	Electroporation CRISPR/Cas9	*CCR5* gene	Genome editing Cas9 RNPs	[65]
Mouse zygotes	Electroporation CRISPR/Cas9	Tyrosinase gene	Genome editing Cas9 RNPs	[180]
Zebrafish (*Danio rerio*) strain AB	Microinjection into embryos CRISPR/Cas9	*akt2* gene	Functional role of *akt2* gene in zebrafish	[181]
Plants				
*Nicotiana tabacum* (tobacco)	Protoplast transformation TALEN	ALS gene/YFP gene	Successful targeted gene replacement with TALENs	[51]
*Arabidopsis thaliana*	*Agrobacterium*-mediated transformation CRISPR/Cas9	*ADH1*, *TT4*, *RTEL1* gene/*bar* gene	Genome editing using CRISPR/Cas9-based nucleases and nickases	[101]
*Arabidopsis thaliana*	*Agrobacterium*-mediated transformation CRISPR/Cas9	*GFP* gene	Stable inheritance of Cas9/sgRNA-generated mutant genes in T2 and T3 progeny	[182]
*Camelina sativa*	*Agrobacterium*-mediated transformation CRISPR/Cas9	*FAD2* gene	Induced mutations caused change in fatty acid composition	[183]
Maize Hi-line	Particle bombardment CRISPR/Cas9	*LIG*, *ALS2*, *MS26* and *MS45*/*MOPAT*-*DSREB*	Genome editing Cas9 RNPs	[184]
Wheat embryos	Particle bombardment CRISPR/Cas9	*TaGW2* gene	Genome editing Cas9 RNPs	[185]
Cyanobacteria				
*Synechococcus elongatus* UTEX 2973	Conjugation CRISPR/Cas9	*nblA*	Genome editing using CRISPR/Cas9-based nucleases	[186]
*Synechococcus elongatus* PCC 7942	Conjugation CRISPR/Cas9	*glgc*/Gm^R^ gene	Increase of succinate Production	[187]
*Synechococcus* UTEX 2973	Conjugation CRISPR/Cpf1	*psbA1*, *nblA*	Genome editing using CRISPR/Cpf1-based nucleases	[188]
*Synechocystis* 6803 *Anabaena* 7120	Conjugation CRISPR/Cpf1	*nblA* *nifH*	Genome editing using CRISPR/Cpf1-based nucleases	[188]
Microalgae				
*C.* strain CC-4350	Glass beads ZFN	*COP3* gene/*aphVIII* gene	Targeted gene knockout induced by ZFN	[58]
*P. tricornutum* CCMP2561	Bombardment TALEN	UGPase/*NAT* gene	Increase in triacylglycerol accumulation	[91]
*C. reinhardtii* CC503	Electroporation CRISPR/Cas9	*FKB12* gene	First application of CRISPR/Cas9 in microalgae	[111]
*P. tricornutum* CCMP2561	Bombardment CRISPR/Cas9	CpSRP54 gene/*Shble*	First application of CRISPR/Cas9 in diatoms	[189]
*C. reinhardtii* CC-124	Electroporation CRISPR/Cas9	*MAA7*, *CpSRP43* and *ChlM*	Targeted gene knockout and knock-in via NHEJ in *Chlamydomonas*	[60]
*C. reinhardtii* CC-4349	Electroporation CRISPR/Cas9	*ZEP* and *CpFTSY*	Production of two-gene knockout mutant	[112]
*C. reinhardtii* CC-400	Glass beads CRISPR/dCas9	*PEPC1* and *RFP*	CRISPRi in *Chlamydomonas*	[104]
*N. oceanica* IMET1	Electroporation CRISPR/Cas9	Nitrate reductase gene/*HygR*	Targeted gene knockout in *Nannochloropsis*	[113]
*N. gaditana* CCMP1894	Electroporation Cas9 Editor line	ZnCys TF BSD	Knockout and attenuation of ZnCys in *Nannochloropsis*	[114]

*ADH1*, alcohol dehydrogenase 1; *akt2*, AKT serine/threonine-protein kinases 2; ALS, acetolactate synthase; *aphVIII*, aminoglycoside 3′-phosphotransferase; *ApoE*, apolipoprotein E; *bar*, herbicide bialaphos; BSD, blasticidin S deaminase; *CCR5*, C–C motif chemokine receptor 5; *ChlM*, Mg-protoporphyrin IX *S*-adenosyl methionine *O*-methyl transferase; *COP3*, light-gated proton channel rhodopsin; *CpFTSY*, signal recognition particle receptor protein, chloroplast; *CpSRP43*, chloroplast signal recognition particle 43; CpSRP54, chloroplast signal recognition particle 54; DHFR, dihydrofolate reductase; DsRed, red fluorescent protein; EGFP, green fluorescent protein; *FAD2*, fatty acid desaturase 2; *FKB12*, peptidyl-prolyl *cis*–*trans* isomerase; *glgc*, glucose-1-phosphate adenylyl transferase; Gm^R^, gentamycin-resistance gene; *HygR*, hygromycin resistance; *LIG*, liguleless1; *MAA7*, beta subunit of tryptophan synthase; MFN2, mitochondrial GTPase mitofusin-2; *MS26* and *MS45*, male fertility genes; *NAT*, *N*-acetyl transferase; *nblA*, phycobilisome degradation protein; *nifH*, nitrogenase reductase; *PEPC*, phosphoenolpyruvate carboxylase; *PsbA1*, D1 protein of photosystem II; *RFP*, red fluorescent protein; *RTEL1*, regulator of telomere length 1; *TaGW2*, gene related to grain development; *TT4*, transparent testa 4; UGPase, UDP-glucose pyrophosphorylase; YFP, yellow fluorescent protein; ZEP, zeaxanthin epoxidase

ZFN and TALEN appeared as an alternative to gene targeting via HR and have been used for targeted modification of genomes [49–52]. They are fusion proteins of the restriction enzyme *Fok*I [53] and their respective DNA binding proteins of zinc finger [54–58] and transcription activator-like effector (TALE) [59], summarized in Fig. 1a. The resulting DSBs induced by *Fok*I are repaired mostly by the error-prone repair mechanism, NHEJ, in most eukaryotes, and mutations can be created at the cleavage sites in the form of small insertions or deletions (INDELs). A donor DNA can be included in the mutagenesis process and can be inserted at the DSB site via NHEJ or HR, which is called a knock-in [60]. A knock-in can be used for more efficient disruption of the target gene or stable expression of a gene at a specific location of the genome [45, 61], which will be discussed in more detail.

CRISPR/Cas9 has gained much attention not only from biologists who are actually working on it but also from the social media including the economic, legal, and industrial sectors [62], which is reflected by the heated legal battles for the patent of CRISPR/Cas9 [63, 64]. This unprecedented attention is mainly due to its excellent potential as the next generation genome editing technique. CRISPR/Cas9 is simple, accurate and efficient compared to other editing techniques [7, 61]. In addition, recombinant Cas9 protein can be assembled with single guide RNA (sgRNA) and delivered as ribonucleoproteins (Cas9 RNPs) into the cells [60, 65]. Delivery of Cas9 RNPs can minimize off-targeting and thus cytotoxicity, and avoid the hassles of cloning markers and sgRNAs. More importantly, the Cas9 nuclease activity can be assessed prior to the lengthy transformation process [60, 65, 66]. Cas9 RNPs, in contrast to vector-driven expression of Cas9 and sgRNAs, may also avoid conflicts from genetically modified organisms (GMOs) depending on the different legal systems [67–69]. Given the advantage of CRISPR/Cas9, this review will focus on it as the choice of genome editing techniques in microalgae. Lately many variations of different classes and types of CRISPR/Cas9 have been reported [70], and thus, CRISPR will be reserved for the general term for all or any variations.

## Biology and application of CRISPR

**Fig. 2:**
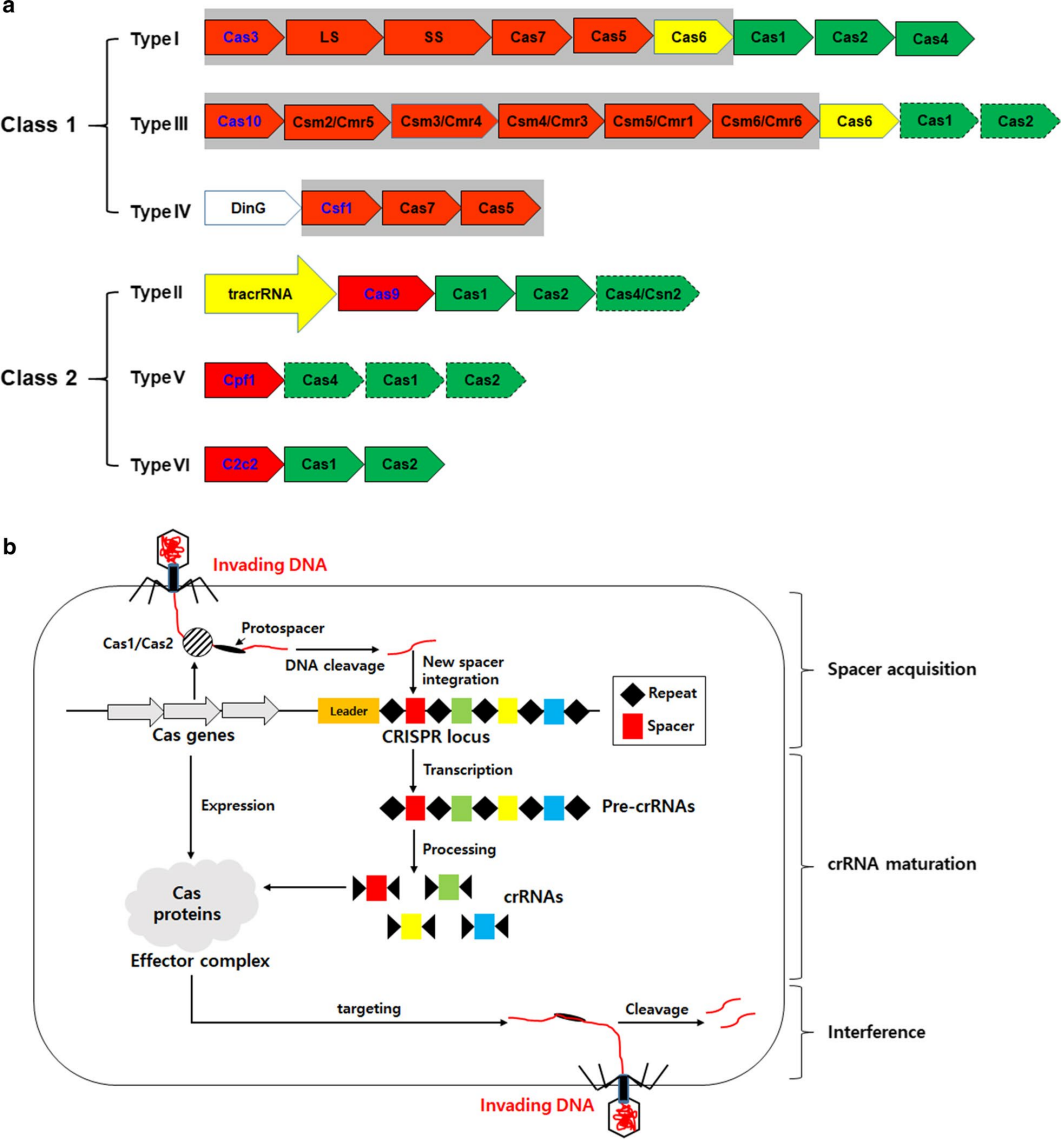
Different subtypes of the CRISPR systems (**a**) and their biological mechanisms of immunity against invading viruses (**b**). Genes involved in interference are shown in the red boxes and those involved in crRNA biogenesis and adaption in the yellow boxes and green boxes, respectively, mainly based on [74, 80]. The signature gene of each types is indicated by blue letters, and the complex of multiple effector proteins is indicated by gray boxes. Dispensable genes are indicated by dashed lines. LS, large subunit; SS, small subunit

The CRISPR locus was first identified as short direct repeats interspaced with short sequences in *E. coli* [71] and later in other bacteria and even in mitochondria and giant viruses, as summarized in Fig. 2a [72, 73]. The CRISPR systems are adaptive immune systems that provide sequence-specific protection against invading viruses or conjugative plasmids [70, 74–76]. It should be noted that there is another type of immunity in bacteria called restriction–modification systems [75, 77], for which the restriction enzymes revolutionized molecular biology resulting in the Nobel Prize in 1978. CRISPR is also revolutionizing all aspects of biology and biotechnology and may be nominated for a Noble Prize [78].

The CRISPR immunity is divided into three stages (Fig. 2b): spacer acquisition (or adaptation), CRISPR RNA (crRNA) biogenesis, and interference stages. During the spacer acquisition stage, a target DNA sequence, known as a protospacer, is excised and inserted at the 5′ end of the CRISPR array producing a new spacer. The subsequent crRNA biogenesis includes transcription and processing of the CRISPR array into mature crRNAs. At the final interference stage, crRNAs guide the effector complex to the target site and cleave the DNA producing DSBs in the re-invading viruses. There are excellent reviews on the biology of CRISPR [70, 74, 76]. This review will focus on the effectors including endonucleases because these nucleases are used for genome editing [79].

The CRISPR systems can be classified into two classes and six types, summarized in Fig. 2a. Class 1 CRISPR systems contain effectors composed of multi-subunit proteins, while those of class 2 contain a single effector with multi-domain such as Cas9 or Cpf1. Class 1 is divided into types I, III and IV, and class 2 includes types II, V and VI, for which the types were numbered based on their order of discovery [74, 80]. The CRISPR systems are very diverse, and Fig. 2 depicts only a representative composition of genes at the CRISPR loci. It is estimated that CRISPR is present in about 50% of bacteria and ~ 90% of archaea [80]. For the purpose of technological applications, class 2 effector nucleases are mainly used due to their convenience in cloning and delivery into host cells. Class 2 nucleases are also diverse in their structural and functional aspects, and this diversity heralds a new age of genome editing that can be customized for individual research projects [74].

### Use of CRISPR for genome editing

Since the initial finding of the mysterious repeats of the CRISPR array in *E. coli* in 1987 and in many other bacteria in 2000 [71, 72, 81], CRISPR/Cas9 has been shown to target DNA specifically in vitro resulting in DSBs [82, 83]. This led to the first reports of genome editing in eukaryotic cells in 2013 [84, 85], and then, an explosive number of reports followed [79] (Table 2).

The advantages of the CRISPR system for genome editing reside in the effector nucleases, for which the nucleases do not require the tedious and labor-intensive cloning of DNA binding domains for targeting specificity. In contrast to the predecessor nucleases ZFN and TALEN, the DNA specificity of CRISPR nucleases is provided by a guide RNA composed of crRNA and trans-activating crRNA (tracrRNA), which were further simplified by the sgRNA [7]. This simple design and preparation of CRISPR enables multiplexed mutagenesis by simply adding up sgRNAs [86, 87]. In addition, CRISPR nucleases appear to be more efficient and more precise compared to predecessors [88].

Another advantage of CRISPR is the diverse nucleases which can be customized for individual needs. Cas9 from *Streptococcus pyogenes* (SpCas9) was initially used for CRISPR-mediated genome editing in animal cells [82, 83]. Lately, nucleases including CRISPR from *Prevotella* and *Francisella* 1 (Cpf1) and CRISPR from *Campylobacter jejuni* (CjCas9) have been introduced for genome editing with improved efficiency and specificity [41, 89, 90]. New class 2 CRISPR systems are being reported, and the number is increasing [74], and different types of nucleases will offer customization of the editing technique for individual research projects.

Best of all, CRISPR appears to be most efficient in microalgal genome editing based on the number of papers reported so far, even though it debuted last in the genome editing field (Table 2). ZFN and TALEN have been used for mutagenesis of *Chlamydomonas* and a diatom, respectively [58, 91], and to the best of our knowledge, there have not been any follow-up reports. Fortunately, CRISPR is gaining a strong foothold in microalgal genome editing, which may provide the possibility of practical and efficient genome editing in microalgae.

However, CRISPR technology still has some limitations, which requires improvements for proper use in genome editing. CRISPR-induced mutations occur randomly depending on the repair of DSBs mostly via NHEJ [92, 93]. Currently precision genome engineering is emerging for better management of mutagenesis, including gene replacement, multiple cleavage and base correction [46], some of which will be further described in “Applications of the CRISPR system” section. Off-targeting can still be an issue for medical and agricultural purposes, even though CRISPR is considered as the most accurate genome editing technique [94]. Fortunately, CRISPR offers a variety of nucleases with improved versatility and/or fidelity, which can also provide optional PAM sequences [41, 89, 90, 95]. These improvements will benefit both biology and biotechnology fields, particularly for microalgal community.

### Technical aspects of CRISPR-mediated genome editing

A key to successful genome editing is efficient delivery of genetic materials, and it has been the main bottleneck in transformation of microalgae. In general, the cell wall is considered as the most significant barrier for the introduction of macromolecules into plant cells [16]. To avoid this problem, the cell wall is removed, and the resulting protoplasts are subjected to PEG-mediated transfection, which appears to be very effective without the need for expensive supplies and equipment [18, 30, 96]. With the proper removal of the cell wall, this technique can result in a transfection efficiency of up to 70% in cassava mesophyll protoplasts [97], which may offer an opportunity to improve microalgal transformation. In fact, as summarized in Table 1, there have been a few reports on PEG-mediated transformation of microalgae including *Chlorella* [26, 27, 29] and *Cyanidioschyzon* [28]. These attempts did not result in a greatly improved transformation efficiency; however, this technique can be improved by complete and/or efficient removal of the cell wall, which may also improve genome editing in microalgae.

Next, successful genome editing in microalgae can be achieved by proper use of the CRISPR nucleases, particularly with the class 2, which are single peptides containing all the functional domains necessary for sequence-specific DNA cleavage [74]. The founding member of such nucleases is SpCas9, and its homologs have been identified in many bacterial strains, and they use two RNA molecules (crRNA and tracrRNA) or one sgRNA for binding to the protospacer, the target sequence on DNA. SpCas9 has the protospacer adjacent motif (PAM) as an additional sequence specificity that provides security minimizing off-target effects. PAM for SpCas9 is mainly NGG but sometimes NAG and is located directly 3′ to the protospacer [7, 79].

Cas9 homologs are equipped with two endonuclease domains producing DSBs, namely the RuvC and HNH domains. These domains are modular enabling individual engineering for different purposes. RuvC was originally identified as part of the *RuvABC* operon (for “resistance to UV light”) and is the endonuclease involved in the resolution of the Holliday junction during UV repair [98]. The HNH domain (named for the histidine, asparagine and histidine residues critical for the nuclease activity) is found in many endonucleases including restriction enzymes and meganucleases [99]. Both RuvC and HNH domains are required for producing DSBs. However, their catalytic sites can be modified to produce a nickase by making an individual mutation of either D10A or H840A, respectively [79, 100]. Such Cas9 nickases are not efficient for inducing mutations but can be used for enhanced knock-in of expression cassettes via HR [101]. In addition, both catalytic sites can be mutated to produce the dead Cas9 (dCas9), and this can be used for variants of CRISPR/Cas9 techniques including CRISPR interference (CRISPRi), where dCas9 is used as a sequence-specific DNA binding protein leading to interference of transcription of the target gene [102–104].

The CRISPR system also offers different types of nucleases, such as Cpf1 and C2c1, which is considered the biggest advantage compared to other genome editing techniques [74]. Cpf1 was initially identified as a type V CRISPR effector from *Prevotella* and *Francisella*, which shows endonucleolytic activities different from Cas9. The differences include a T-rich PAM site and staggered cleavage of DNA located 3′ to PAM [95]. Cpf1 from *Acidaminococcus* sp. (AsCpf1) and *Lachnospiraceae bacterium* (LbCpf1) have been used for genome editing in animals and plants [66, 89, 95]. These Cpf1s do not require tracrRNA, which offers simpler preparation of the guide RNA. In addition, it appears to be more efficient and accurate than Cas9 [66, 89] and thus has emerged as the next generation nuclease for genome editing. Additional type V nucleases, but less studied, include C2c1 and C2c3. These type V effectors are further classified as subtype V-B and V-C, while Cpf1 belongs to the V-A subtype [74]. These subtypes are characterized by different domain structures, which can be used for customized genome editing purposes.

Interestingly, there is another type of CRISPR system containing endonucleases that cleave RNA targets instead of DNA. These belong to the type VI, and the sequence-specific RNases include C2c2 and many others [74, 105]. The type VI CRISPR system is reminiscent of the eukaryotic RNA silencing mechanism involving the RNA-induced silencing complex (RISC), in which Argonaute (AGO) and Dicer carry out guide RNA (i.e., siRNA)-based sequence-specific identification and cleavage of the target RNA, respectively [106–108]. However, C2c2 carries both functions [109] revealing another bacterial ingenuity of simplicity, in contrast to the bulky, complex and elaborate eukaryotic counterparts. RNAi in eukaryotic systems is not very reliable in the suppression of gene expression particularly in microalgae [110], and the type VI CRISPR systems may provide a better alternative.

Different from any gnome editing techniques, the CRISPR system enables the delivery of preassembled Cas9 or Cpf1 RNP with the cognate guide RNAs in vitro [65, 89]. Compared to vector-driven expression of the nucleases and guide RNAs, the RNP system is simple and convenient without the need for the laborious and time-consuming cloning process and thus obtains results faster. There are other benefits of the RNP approach including the pre-test of the nuclease activity in vitro. There was a correlation between in vitro and in vivo activities of SpCas9 in *Chlamydomonas* for different target sites in the same gene [60]. In addition, RNP delivery can minimize off-target effects and possible toxicity from the continuous expression of a nuclease [60, 65, 111]. It does not introduce any artificial DNA including markers and heterologous genes and may avoid GMO conflicts [67]. Best of all, it has been successful in the recalcitrant model algae *Chlamydomonas* [60, 112]. This may provide interesting opportunities to deal with the difficulties in microalgal genome editing.

Considering the difficulties in delivering genetic materials into microalgal cells, microalgal genome editing is gaining momentum with the CRISPR/Cas9 systems summarized in Table 2. In case of vector-based Cas9 expression in microalgae, codon usage can be optimized for better expression of Cas9 in microalgae [60, 111–114]. This achievement is in part due to the efficiency and simplicity of the CRISPR systems [60, 112]. Another reason can be due to the fact that many microalgae are haploids, enabling the selection of knocked-out clones without the need to make homozygotes. Green algae, including chlorophytes and charophytes, are considered to have a haplontic life cycle in which their genomes are haploids during vegetative growth [115], and this may be true in other microalgae including *Nannochloropsis* [116] and *Guillardia* [117]. On the other hand, haploids do not allow the knockout of essential genes, which should be considered before making a gene list for potential knock-outs. In this case, one can consider the CRISPR knockdown approaches such as CRISPRi and attenuation of gene expression by targeting UTRs that will be described in more detail [118, 119].

## Applications of the CRISPR system

The simplicity of the CRISPR systems has led to a sudden increase in variant technologies, which was difficult with previous techniques of genome editing or any reverse genetic techniques. First, the Cas9 nuclease can be easily manipulated to create nickases or dCas9, and these variants can be used for additional genome manipulation including knock-in and CRISPRi [7, 60, 102]. Second, multiple sites can be targeted simultaneously by simply adding guide RNAs, for which two sites can be targeted to obtain a chromosomal deletion, inversion or translocation [7]. The numbers can be increased to target multiple genes at the same time [86] or even to create a barcoded CRISPR mutant library [87]. It should also be noted that anti-CRISPRs have been identified in bacteriophages as an arms race against their hosts [120, 121], which provides an interesting possibility that they can be used in genome editing, such as a conditional knockout. There is a long list of applications for the CRISPR systems [119, 122, 123], and some of these that are relevant or applicable to microalgae will be described in this review.

### dCas9 for CRISPRi and manipulation of gene expression

Cas9 contains well defined endonuclease domains that can be modified to create a nickase or even dCas9, and these mutants can also be used for new exciting techniques. In particular, dCas9 can bind to the target site without cleaving DNA, and this can interfere with cellular processes including transcription. This CRISPRi technique has been shown in bacteria and even in *Chlamydomonas* [102, 104, 119, 124]. In bacteria, dCas9 interferes with the expression of target gene(s) by providing steric hindrance to RNA polymerase or transcription factors, depending on the location of target sites [102, 124]. In *Chlamydomonas*, CRISPRi is shown to knockdown the expression of phosphoenolpyruvate carboxylase (PEPC) [104], albeit less effective possibly due to the difference between prokaryotic and eukaryotic transcription mechanisms, where eukaryotic transcription is more tolerant to DNA binding proteins including chromatin.

**Fig. 3:**
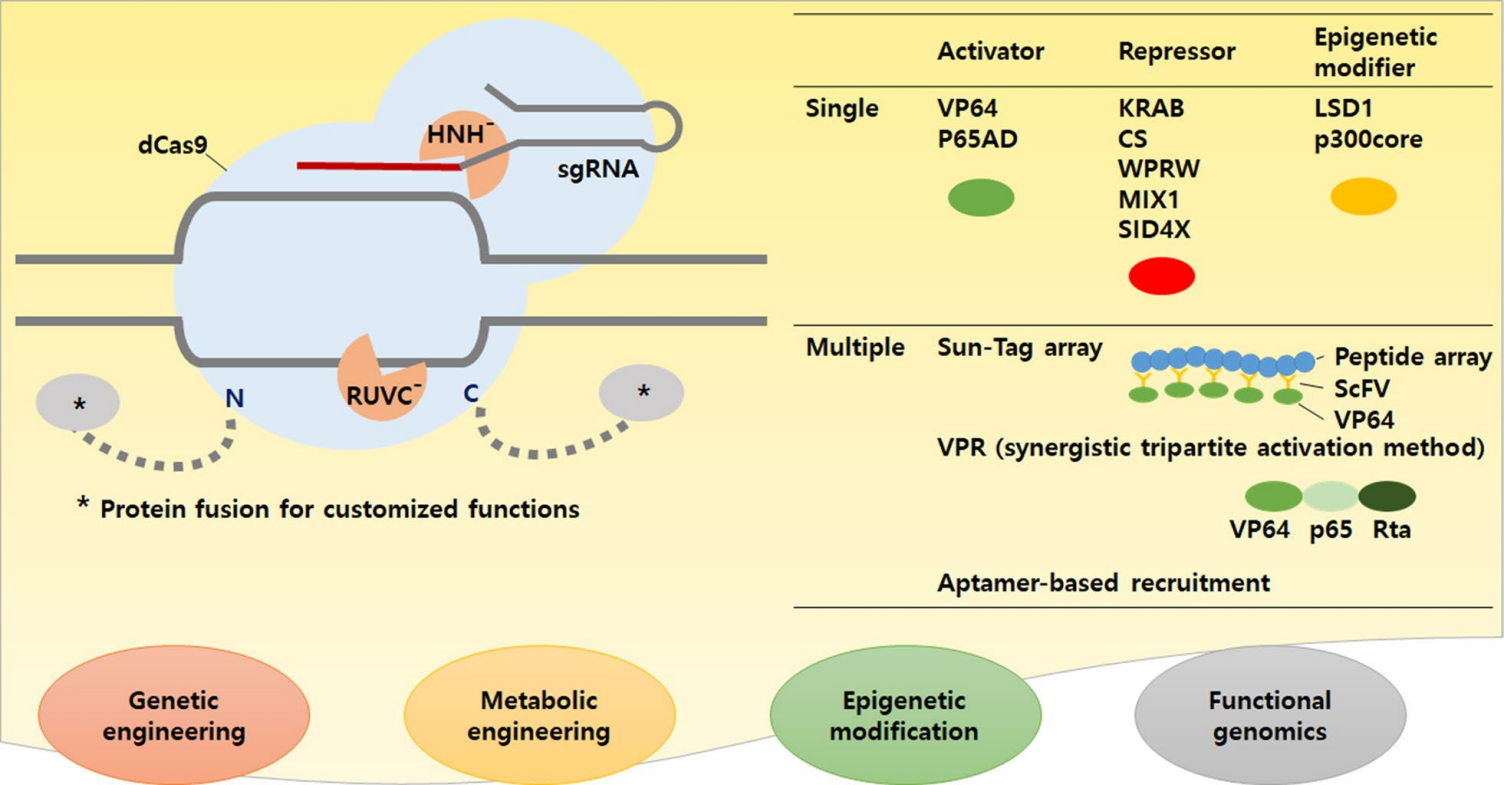
Application of the CRISPR system for manipulation of gene expression using dCas9. Different functional domains of transcriptional regulators can be fused to dCas9, which result in activation or repression of the target genes

dCas9 can also be repurposed for other functions by fusion of domains involved in transcriptional activation (CRISPRa), repression (CRISPRi), and epigenetic regulation (Fig. 3). It should be noted that CRISPRi is used for simple interference without any fused proteins [102, 124] and for active interruption with repressor domains [119], which may be resolved in the future. For CRISPRa, the multiple repeats of the herpes simplex VP16 activation domain (VP64) and the nuclear factor-κB transactivating subunit activation domain (p65AD) have commonly been used as activator domains in eukaryote systems [125–127]. These subunits are fused to the N or C terminus of dCas9 as a single or multiple units. After it is shown that having more activators improve the activation efficiency, several units including different activator domains can be added. For example, the ‘SunTag’ array consists of 10 copies of a small peptide epitope each linked with VP64 and sfGFP by scFV [128]. As another example, the synergistic tripartite activation method (VPR) uses a tandem fusion of three transcription activators, VP64, p65 and Rta [129]. The synergistic activation mediator (SAM) is fused to VP64, and two MS2 RNA aptamers added to the tetraloop and second stem-loop of the sgRNA recruit p65Ad and heat shock factor1 (HSF1) through MCP [130, 131].

Repressors that have been used in CRISPRi include MAX-interacting protein 1 (MXI1) from yeast, Krüppel-associated box (KRAB) domain of Kox1, the CS domain of HP1α, the WPRW domain of Hes1, or four concatenated mSin3 domains (SID4X) which are fused either to the amino or carboxyl terminus [125, 132, 133]. However, it appears that this field has some room for improvements. Epigenetic regulation is critical for proper expression of genes, which can be achieved by histone and DNA modifications. The Lys-specific histone demethylase 1 (LSD1) fusion protein, the catalytic core of histone acetyltransferase (p300), or DNMT3A, a DNA methyltransferase, has been tested with dCas9 [134–136]. These can be used as epigenome editing tools to reveal interactions between the epigenome and regulatory elements and their epigenetic mechanism of gene expression [137, 138] not only in higher eukaryotes but also in microalgae.

### Knock-ins with CRISPR

CRISPR can also be used for a knock-in and replacement of a gene(s), if given a donor DNA(s) (Fig. 1). During the repair process of DNA breaks caused by CRISPR nucleases, the donor DNA can be integrated at the cleavage site via HDR or NHEJ. The donor DNA without any inserted expression cassette can also be used for replacement of a gene via HDR [7, 139]. Given the homologous sequences flanking the transgene, single-stranded donor DNAs can be integrated at the cleavage site of nucleases including TALEN [140, 141]. Alternatively, Cas9 nickase (D10A) can also enhance knock-in or gene replacement in plants [101]. Interestingly, a knock-in can also occur through NHEJ (Fig. 1a), where no homologous sequences are present in the transgene. NHEJ-mediated knock-in events have been reported in zebrafish and *Chlamydomonas* [60, 142].

Knock-in events can be used in targeted integration of transgenes at certain locations on the genome. Random integration of transgenes suffers from position effects and transgene silencing [143, 144]. Such detrimental effects are also known in microalgae including *Chlamydomonas* [33, 34, 145–147] and can influence the stable expression of transgenes. These problems can be solved in part by integrating transgenes at transcriptionally favorable locations on the genome. Actually, such “safe-harboring” has been shown; the knock-in of transgenes at transcriptionally active sites, e.g., near the rDNA cluster, increases the expression of transgenes [46, 148, 149]. Currently, functional genomic data including RNA-Seq and epigenomics mapping of histone modifications can offer candidates of transcriptionally active locations. These locations can be targeted for the integration of expression cassettes. In microalgae, fortunately, cloning of the flanking homologous sequences may be not necessary because the knock-in via NHEJ can occur in *Chlamydomonas* and *Nannochloropsis* [60, 114].

## Perspectives of genome editing in microalgae

Microalgae and CRISPR are relatively new additions to biotechnology fields, which are expected to contribute to biomaterial production and genome editing techniques, respectively. Combination of the two quintessential components is potentially the key to solve the environmental problems associated with usage of fossil fuels. Such example has been reported recently, where CRISPR-induced knockout or attenuation of a regulatory gene can increase lipid accumulation in industrial microalgae *Nannochloropsis* [114].

Different from previous editing techniques, CRISPR allows systemic, albeit labor-intensive, screening of knockout mutations due to its simplicity and convenience [113, 114]. This is reflected by the number of reports, in which ZFN and TALEN-induced mutagenesis for only one for each techniques since 2013 [58, 91]. However, successful genome editing with CRISPR alone has been documented three times in 2016 as summarized in Table 2. This success heralds new and improved genome editing field in microalgae, which attracts great interests of academic and industrial biology and biotechnology.

Even though microalgae are difficult in genetic manipulation, their biological characteristics offer advantages. They are single cells, and mostly contain haploid genomes for their vegetative cells [115–117, 150]. This leads to convenient knockout without the necessity of regeneration, which is considered a main bottleneck in plants [15]. In addition, being haploid, microalgae do not have to go through another generation for homozygotes. On the other hand, complete knockout of an essential gene is not possible, in which attenuation should be considered. For example, attenuation can be achieved by targeting outside of coding sequence such as untranslated regions or by CRISPRi [114, 119].

### Problems and possible solutions with CRISPR application in microalgae

Microalgae are still difficult to manipulate genes possibly due to their multitude of problems. Firstly, it is hard to deliver genetic materials into the cells, probably because they have unique cell wall and surface structures that reflect their complex taxonomic lineages [151]. This diversity hinders development of standardized protocols for transformation. To avoid such problems, one can remove the cell wall and employ protoplast transformation, which has been demonstrated for a few microalgae including *Chlorella* as summarized in Table 1 [26, 27, 29]. Protoplasts are in general easier to transform, which may improve efficiencies of not only transformation but also genome editing. Secondly, microalgae may have very efficient silencing systems against introduced genetic materials including DNA and RNA at the transcriptional and post-transcriptional levels. Such silencing systems have been reported for the model algal *Chlamydomonas* [33, 145], and are expected to exist in other microalgae [34]. Temporary knockdown of one of the silencing components may improve transformation efficiency [34]. Permanent mutations of silencing components are not recommended, because they are also involved in genome stability [145].

Cas9 RNP appears to be more efficient than vector-driven Cas9 in *Chlamydomonas* [60, 111, 112], and is advantageous if heterogeneous genetic material should not be introduced, particularly in areas where GMOs are prohibited. However, high quality non-toxic recombinant Cas9 protein is not easy to prepare or is expensive to purchase from a company. However, for research purposes, a stable line of Cas9 or equivalent nucleases can be constructed for efficient gene editing. For example, the Cas9 Editor line has been successfully employed to produce 18 mutations in *Nannochloropsis* [114].

Precision genome editing technologies require precise mutagenesis without producing off-targeting events, which has not been well established in microalgae. Such precision is crucial for certain applications of CRISPR particularly for gene therapy in human, and is well established in animals and plants [152]. Fidelity of genome editing can be improved by Cas9 RNP in animal cells [65, 66], and Shin et al. reported no off-targeting events in *Chlamydomonas* using Cas9 RNP [60]. Other than the latter, off-targeting has not been examined in microalgae, where such efforts should improve safety and consistency of genome editing in microalgae.

## Conclusions

Genome editing is essential for obtaining mutations of target genes enabled by recombinant nucleases with sequence specificity. The latest nucleases found in the CRISPR systems are far better than the predecessors in terms of their simplicity, accuracy and efficiency. This improved CRISPR technology can be used in the correction of mutations, replacement of genes, and targeted integration of overexpression cassettes. It can also be used for many other purposes including attenuation of gene expression, removal of transgenic markers, etc., and the list is getting longer. The microalgal community is catching up with this new and exciting technology but is lagging behind the main stream technical developments in animals and plants. We need to first solve the fundamental problems in microalgae, which is the inefficient delivery of genetic materials into the cell. Given such a tremendous barrier, many more papers have been reported with CRISPR compared to the previous techniques, which may herald a new age of genome editing in microalgae.

## Abbreviations

amiRNAs: artificial microRNAs
Cas9 RNPs: Cas9/gRNA ribonucleoproteins
Cpf1: CRISPR from *Prevotella* and *Francisella* 1
CRISPR/Cas9: clustered regularly interspaced palindromic sequences/CRISPR-associated protein 9
CRISPRa: CRISPR activation
CRISPRi: CRISPR interference
crRNA: CRISPR RNA
dCas9: dead Cas9
DSBs: double strand breaks
GMO: genetically modified organism
GT: gene targeting
HDR: homology-dependent recombination
HR: homologous recombination
INDELs: insertions or deletions
miRNAs: microRNAs
NHEJ: non-homologous end joining
PAM: protospacer adjacent motif
PEG: polyethylene glycol
RNAi: RNA interference
sgRNA: single guide RNA
siRNA: small interfering RNAs
SpCas9: Cas9 from *Streptococcus pyogenes*
TALEN: TAL effector endonuclease
tracrRNA: trans-activating crRNA
VP64: VP16 activation domain
ZFN: zinc-finger nuclease
